# S1P/S1PR1 signaling is involved in the development of nociceptive pain

**DOI:** 10.3389/fphar.2024.1407347

**Published:** 2024-07-09

**Authors:** Daosong Dong, Xue Yu, Xueshu Tao, Qian Wang, Lin Zhao

**Affiliations:** ^1^ Department of Pain, The First Hospital of China Medical University, Shenyang, China; ^2^ Key Laboratory of Precision Diagnosis and Treatment of Gastrointestinal Tumors (China Medical University), Department of Surgical Oncology and General Surgery, The First Hospital of China Medical University, Ministry of Education, Shenyang, China; ^3^ Medical Oncology, Department of Gastrointestinal Cancer, Liaoning Cancer Hospital and Institute, Shenyang, China

**Keywords:** nociceptive pain, S1P, S1PR1, microglia, astrocytes, inflammation

## Abstract

**Background:**

Pain is a complex perception involving unpleasant somatosensory and emotional experiences. However, the underlying mechanisms that mediate its different components remain unclear. Sphingosine-1-phosphate (S1P), a metabolite of sphingomyelin and a potent lipid mediator, initiates signaling via G protein-coupled receptors (S1PRs) on cell surfaces. It serves as a second messenger in cellular processes such as proliferation and apoptosis. Nevertheless, the neuropharmacology of sphingolipid signaling in pain conditions within the central nervous system remains largely unexplored and controversial.

**Methods:**

Chronic nociceptive pain models were induced in vivo by intraplantar injection of 20 μL complete Freund's adjuvant (CFA) into the left hind paws. We assessed S1P and S1PR1 expression in the spinal cords of CFA model mice. Functional antagonists of S1PR1 or S1PR1-specific siRNA were administered daily following CFA model establishment. Paw withdrawal response frequency (PWF) and paw withdrawal latency (PWL) were measured to evaluate mechanical allodynia and thermal hyperalgesia, respectively. RT-PCR assessed interleukin (IL)-1β, IL-6, and tumor necrosis factor (TNF)-α levels. Western blotting and immunofluorescence were used to analyze glial fibrillary acidic protein (GFAP), ionized calcium-binding adapter molecule (Iba1), STAT3, ERK, and p38 MAPK protein expression.

**Results:**

In the chronic nociceptive pain model induced by CFA, S1P and S1PR1 expression levels were significantly elevated, leading to activation of spinal cord glial cells. S1PR1 activation also promoted MMP2-mediated cleavage of mature IL-1β. Additionally, S1PR1 activation upregulated phosphorylation of STAT3, ERK, and p38 MAPK in glial cells, profoundly impacting downstream signaling pathways and contributing to chronic nociceptive pain.

**Conclusion:**

The S1P/S1PR1 axis plays a pivotal role in the cellular and molecular mechanisms underlying nociceptive pain. This signaling pathway modulates glial cell activation and the expression of pain-related genes (STAT3, ERK, p38 MAPK) and inflammatory factors in the spinal dorsal horn. These findings underscore the potential of targeting the S1P system for developing novel analgesic therapies.

## 1 Introduction

Nociceptive pain is the most common type of chronic pain encountered in clinical practice which is primarily triggered by injury and subsequent inflammation of peripheral tissues (Ronchetti et al., 2017). Nociceptive pain is a disorder characterized by persistent nociceptive hypersensitivity, often accompanied by allodynia (pain elicited by non-painful stimuli), and hyperalgesia (an amplified response to painful stimuli) (Descalzi et al., 2015). Nociceptive pain can have a significant negative impact on the patient’s quality of life, in addition to causing significant financial losses (Muley et al., 2016). Common analgesic drugs are the first line of treatment for nociceptive pain. However, the incidence of serious adverse events reduces the efficacy of the drug. Therefore, new reliable and innovative anti-inflammatory analgesics are needed.

Sphingolipids are important components of cell membranes, and ceramide-1-phosphate, sphingosine, and phospho-1-sphingosine (S1P) are important metabolites of sphingolipids, which are involved in signal transduction of various important physiological processes such as growth and apoptosis (Hannun and Obeid, 2018). In response to extracellular stimuli (e.g., nerve growth factor and cytokines), phosphorylation of sphingosine produces the biologically active S1P. The bioactive sphingolipid metabolite S1P is now considered a key regulator that is produced by two sphingosine kinase isoenzymes, SphK1 and SphK2 (Fyrst and Saba, 2010; Perez-Jeldres et al., 2021). S1P can be secreted into the extracellular milieu and generate some of its high biological activity by acting in an autocrine, paracrine and/or endocrine manner (Takabe et al., 2008). S1P initiates the classical G protein coupled receptor (GPCR) signaling pathway by binding to the cell surface homologous receptor (S1PR) and can be used as a secondary messenger to participate in cell proliferation and apoptosis (Wang et al., 2020). Unlike ceramide and sphingosine, it stimulates cell growth and inhibits apoptosis. Abnormal changes in the expression of S1P or the structure of S1PRs can lead to many diseases. For example, S1P participates in the transformation of many kinds of malignant tumors and promotes the growth and propagation of tumors (Degagne et al., 2014; Ratajczak et al., 2014; Ogretmen, 2018). S1P is also involved in the occurrence and development of a variety of autoimmune diseases, such as multiple sclerosis, systemic lupus erythematosus, rheumatoid arthritis, and ulcerative colitis (Chi, 2011; Tsai and Han, 2016; Nielsen et al., 2017; Chun et al., 2021; McGinley and Cohen, 2021; Duan et al., 2022).

Moreover, free S1P can reach a higher concentration locally at sites of injury (Salvemini et al., 2013). S1P or its receptor could be explored and considered as a promising treatment target for tumors, immunity and other diseases (Cui et al., 2022). For example, selective S1PR1 modulators have been approved by the FDA for use in multiple sclerosis (Cohen et al., 2010). Recent studies have also found that S1P is involved in the occurrence and development of pain. For example, S1P and S1P receptor 3 (S1PR3) are key regulators of acute mechanical perception (Hill et al., 2018). S1P also participates in spontaneous pain and thermal pain hypersensitivity (Hill et al., 2018). However, studies on the effect and mechanism of S1P and its receptors on pain are still controversial. Therefore, we constructed a nociceptive pain model induced by CFA to explore the effects of S1P and its receptor S1PR1 on the occurrence and development of pain and to provide new ideas for the future research and development of targeted drugs or foods for the use of S1P and S1PR1 in the field of pain.

## 2 Materials and methods

### 2.1 Animals

C57BL/6 mice (male, 6–8 weeks, 20–25 g) were obtained from Liaoning Changsheng Biological Center, Shenyang, China. The mice were housed in a centralized location and kept under standard laboratory conditions (5 animals per individual cage, 24°C ± 2°C, and 50%–70% humidity under a 12/12-h light/dark cycle) with free access to water and food. The procedures used in the animal experiments were approved by the Animal Care and Ethics Committee of China Medical University (Issue number: KT2022115). Utmost care was taken to ensure the welfare of the rodents, and their usage was kept to a minimum.

### 2.2 Drugs and treatment

Complete Freund’s adjuvant (F5881) was purchased from Sigma‒Aldrich Canada Ltd. S1P (HY-108496, 5 µL, 100 Um), SK1-I (HY-119016), FTY720 (HY-12005), siponimod (HY-12355), and KRP-203 (HY-13660) were purchased from MCE (Mair et al., 2011). Inflammatory pain was induced by intraplantar injection of 20 μL CFA in the left hind paws. For intragastric administration, the mouse was fixed in an upright position, and the gavage needle was gently inserted into the mouth and then the esophagus along the posterior wall of the pharynx to stimulate the mice to swallow. The needle was quickly inserted into approximately 3–4 cm, and the liquid was injected. After intragastric administration, the gavage needle was gently removed to observe whether there was any leakage, and attention was given to the health status of the mice. For intrathecal injection, mice were fixed, and the intervertebral space of L5-L6 was used as the injection position to align the needle along the midline of the spine. The needle was inserted gently vertically, and when touching the bone, the angle was slowly reduced to approximately 30°, the needle was slid into the intervertebral space, and the liquid was injected. Placement was confirmed by a lateral tail flick as the needle entered the subarachnoid space.

### 2.3 Behavioral tests

All behavioral tests were performed in an individual Plexiglas chamber on an elevated metal mesh floor under stable room temperature and humidity. Mice were habituated to the environment for at least 1 h before the experiments. The paw withdrawal response frequency (PWF) was measured to assess mechanical allodynia using Dixon’s updown method. A 0.07- or 0.4-g von Frey filament was applied ten times with 10-s time intervals in the plantar surface of a hind paw until it bent into an S shape for a duration of ≤ 6 s. A positive reaction was recorded as rapid paw withdrawal, licking or shaking of the paws. The percentage of paw withdrawal responses after each filament stimulus was counted as the frequency. The paw withdraw latency (PWL) was used to assess thermal hyperalgesia. Mice were placed on the glass plate of the Hargreaves apparatus. A radiant heat source through a keyhole was applied to the dorsal surface of the hind paw (maximum time of 20 s to eliminate any tissue injury), and the latency to lift, lick, or withdraw the paw was determined for all the mice. All behavioral tests were performed in a blinded manner.

### 2.4 ELISA

Whole blood samples collected in the mouse serum separation tube were placed at room temperature for 2 h and then centrifuged at 1,000 × *g* for 20 min, and the supernatant was obtained for follow-up experiments. The mouse tissues were washed with precooled PBS (0.01 m, pH = 7.4), the residual blood was removed, and the tissues were cut into pieces. The chopped tissue and the corresponding volume of PBS were placed into the grinder to split the tissue cells. The homogenate was centrifuged at 5,000 × *g* for 10 min, and the supernatant was taken for follow-up experiments. The specific determination method was performed according to the ELISA kit (Echelon Biosciences). Fifty microliters of standard working solution or sample was added to each well, and then 50 μL of biotin conjugate working solution was added immediately and incubated at 37°C for 60 min. The liquid was discarded, and 200 μL of washing buffer was added to each well and washed 3 times. After drying, 100 μL HRP enzyme was added to each well and incubated at 37°C for 60 min. The liquid was discarded, and 200 μL of washing buffer was added to each well and washed 5 times. After drying, 90 μL TMB was added to each well and incubated at 37°C for 20 min. Fifty microliters of terminator solution was added to each well, and the 450 nm OD value was read immediately.

### 2.5 Real-time polymerase chain reaction (RT‒PCR)

Total RNA was extracted from ipsilateral hind paw and L4-6 spinal cord tissue using TRIzol reagent (Invitrogen). cDNA was synthesized from total RNA (1 μg) using Hifair II 1st strand cDNA Synthesis SuperMix (Yeasen, China). The reverse transcription procedure was performed at 25°C for 25 min and 42°C for 30 min followed by 85°C for 5 min. The sequences of the PCR primers (Sango Biotech, Shanghai, China) are listed in Table 1. qPCR was carried out at 95°C for 2 min followed by 40 cycles of 95°C for 15 s and 60°C for 1 min. qPCR was conducted using the SYBR-Green System (Yeasen, China). Relative gene expression was calculated using the 2^−ΔΔCT^ method, with GAPDH as the internal control and reference gene.

**TABLE 1:** All primers and other sequences used.

Gene	5′-3′
*il1b-F*	TGC​CAC​CTT​TTG​ACA​GTG​ATG
*il1b-R*	TTC​TTG​TGA​CCC​TGA​GCG​AC
*il6-F*	GCC​TTC​TTG​GGA​CTG​ATG​CT
*il6-R*	TGT​GAC​TCC​AGC​TTA​TCT​CTT​GG
*tnf-α-F*	CAC​TTG​GTG​GTT​TGC​TAC​GA
*tnf-α-R*	CAC​TTG​GTG​GTT​TGC​TAC​GA
*inos-F*	GTT​CTC​AGC​CCA​ACA​ATA​CAA​GA
*inos-R*	GTG​GAC​GGG​TCG​ATG​TCA​C
*il-10-F*	GCT​CTT​ACT​GAC​TGG​CAT​GAG
*il-10-R*	CGC​AGC​TCT​AGG​AGC​ATG​TG
*arg1-F*	CTC​CAA​GCC​AAA​GTC​CTT​AGA​G
*arg1-R*	AGG​AGC​TGT​CAT​TAG​GGA​CAT​C
*s1pr1-F*	ATG​GTG​TCC​ACT​AGC​ATC​CC
*s1pr1-R*	CGA​TGT​TCA​ACT​TGC​CTG​TGT​AG
*s1pr2-F*	ATG​GGC​GGC​TTA​TAC​TCA​GAG
*s1pr2-R*	GCG​CAG​CAC​AAG​ATG​ATG​AT
*s1pr3-F*	ACT​CTC​CGG​GAA​CAT​TAC​GAT
*s1pr3-R*	CAA​GAC​GAT​GAA​GCT​ACA​GGT​G
*s1pr4-F*	GTC​AGG​GAC​TCG​TAC​CTT​CCA
*s1pr4-R*	GAT​GCA​GCC​ATA​CAC​ACG​G
*s1pr5-F*	GCT​TTG​GTT​TGC​GCG​TGA​G
*s1pr5-R*	GGC​GTC​CTA​AGC​AGT​TCC​AG
*gapdh-F*	AGG​TCG​GTG​TGA​ACG​GAT​TTG
*gapdh-R*	TGT​AGA​CCA​TGT​AGT​TGA​GGT​CA

### 2.6 Immunofluorescence

Mice were deeply anesthetized with 1% sodium pentobarbital and transcardially perfused with 20 mL of 0.9% cold saline followed by 4% paraformaldehyde in 0.1 M phosphate-buffered saline (PBS, pH 7.4) via the cardiovascular system. After perfusion, the L5 spinal segments were removed and postfixed overnight at 4°C. The next day, the segments were dehydrated in a sucrose gradient (20% and 30% sucrose in PBS) at 4°C for 24 h until they completely sank to the bottom. Serial frozen 30-µm spinal cord sections were cut on a coronal plane using a cryostat and prepared for immunofluorescence staining. After washing with phosphate-buffered saline, the sections were blocked with 0.1% BSA for 1 h at room temperature, followed by incubation overnight at 4°C with the primary antibodies rabbit anti-S1PR1 (1:200; Proteintech # 55133-1-AP), goat anti-IBA-1 (1:200; Abcam # ab289874), mouse anti-GFAP (1:200; Proteintech # 60190-1-Ig), and mouse anti-NeuN (1:200; Abcam # ab279296) diluted in PBS. The sections were incubated with the corresponding secondary antibodies for 1 h after washing three times with PBS at room temperature. The stained sections were mounted with 4′, 6-diamidino-2-phenylindole (DAPI) (Abcam #ab104139), and images were captured using a fluorescence microscope (Leica).

### 2.7 Western blotting

L4-6 spinal cord tissues were homogenized and lysed on ice for 30 min in 100 μL RIPA lysis buffer solution (Beyotime Biotechnology) containing protease and phosphatase inhibitors. The homogenates were centrifuged at 12,000 rpm for 15 min at 4°C. The protein samples were quantified using the BCA protein assay (Beyotime Biotechnology). The proteins were separated on a 10% SDS‒PAGE gel (Bio-Rad) and transferred to PVDF membranes, which were then blocked with 5% BSA in TBS-Tween. The blots were probed with antibodies against S1PR1 (1:1,000, Proteintech # 55133-1-AP), STAT3 (1:1,000, Cell Signaling # 9139), phosphorylated STAT3 (1:1,000, Cell Signaling #9131), ERK (1:1,000, Cell Signaling #9102), phosphorylated ERK (1:1,000, Cell Signaling #4370), phosphorylated P38 (1:1,000, #4511), p38 (1:1,000, #9212), and GAPDH (1:10,000, Zhongshan Golden Bridge Biotechnology #TA-08). The blots were incubated with horseradish peroxidase-labeled rabbit anti-mouse (1:5,000, Abcam #ab6789) or goat anti-rabbit (1:5,000, Abcam #ab6721) secondary antibodies. The protein bands were visualized with enhanced chemiluminescence reagents (Sigma), and the band densities were analyzed using ImageJ software. All experiments had at least four biological replicates, and representative blots are presented in the figures.

### 2.8 Statistical analysis

The data are expressed as the mean ± standard error of the mean (SEM). Statistical analysis was performed using Prism GraphPad 6.0. For the behavioral tests, the differences between groups were analyzed using two-way repeated-measures ANOVA followed by Bonferroni’s *post hoc* test. For immunofluorescence, RT‒PCR, and Western blotting, differences between groups were compared by one-way ANOVA with Tukey’s *post hoc* test. For all comparisons, *p* < 0.05 indicated statistical significance.

## 3 Results

### 3.1 S1P was involved in nociceptive pain

**FIGURE 1:**
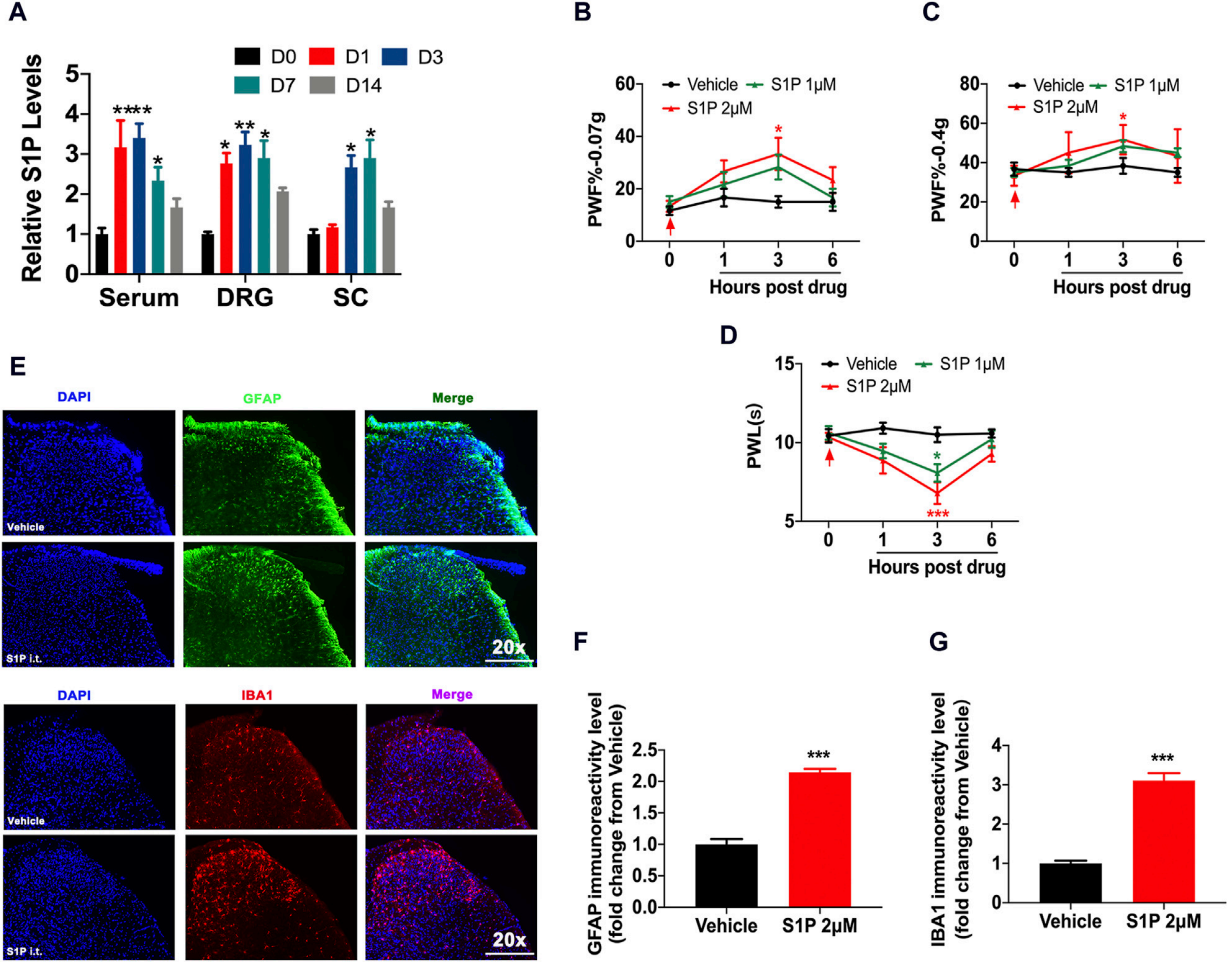
S1P led to nociceptive pain. **(A)** The content of S1P in the serum, DRG and spinal cord of CFA mice at 0, 1, 3, 7, and 14 days was analyzed by ELISA (n = 3 biological repeats, 3 mice/repeat/group). Data are shown as the mean ± SEM. *p < 0.05, **p < 0.01 vs. D0. **(B–D)** PWF and PWL of mice after i.t. injection of S1P (1 μM, 2 μM) compared to vehicle (n = 6 mice/group). Data are shown as the mean ± SEM. *p < 0.05, ***p < 0.001 vs. Vehicle. **(E)** The activation of astrocytes (GFAP: a marker of astrocytes) and microglial cells (IBA1: a marker of microglia) in the spinal dorsal horn was detected by immunofluorescence after S1P injection (n = 3–4 biological repeats). **(F,G)** The immunoreactivity levels of GFAP and IBA1 in the spinal dorsal horn after S1P injection (n = 3–4 biological repeats). Data are shown as the mean ± SEM. ***p < 0.001 vs. Vehicle. The red arrow represents the time of model establishment.

The CFA model was selected as a chronic nociceptive pain model. We found that the content of S1P in the serum of CFA mice was significantly increased, especially on days 1 and 3. In the dorsal root ganglion (DRG), the level of S1P increased from day 1 to day 7, and in the spinal cord, the expression of S1P began to increase 3 days after CFA model induction (Figure 1A). To further verify the role of S1P in pain, exogenous S1P was administered through intrathecal injection, and the mice showed transient mechanical and thermal hyperalgesia (Figures 1B–D). S1P regulates the development of neuroinflammation in the nervous system (Wang et al., 2021). Neuroinflammation is typically characterized by the activation of microglia and astrocytes (Kasatkina et al., 2021). Through immunofluorescence detection, we found that administration of S1P activated microglia and astrocytes in the dorsal horn of the spinal cord in the CFA model, which was closely related to nociceptive pain (Figures 1E–G). These results indicated that pathological pain could cause an increase in S1P expression and that S1P may participate in the occurrence of nociceptive pain.

### 3.2 SPHK1 inhibitor relieved CFA-induced nociceptive pain

**FIGURE 2:**
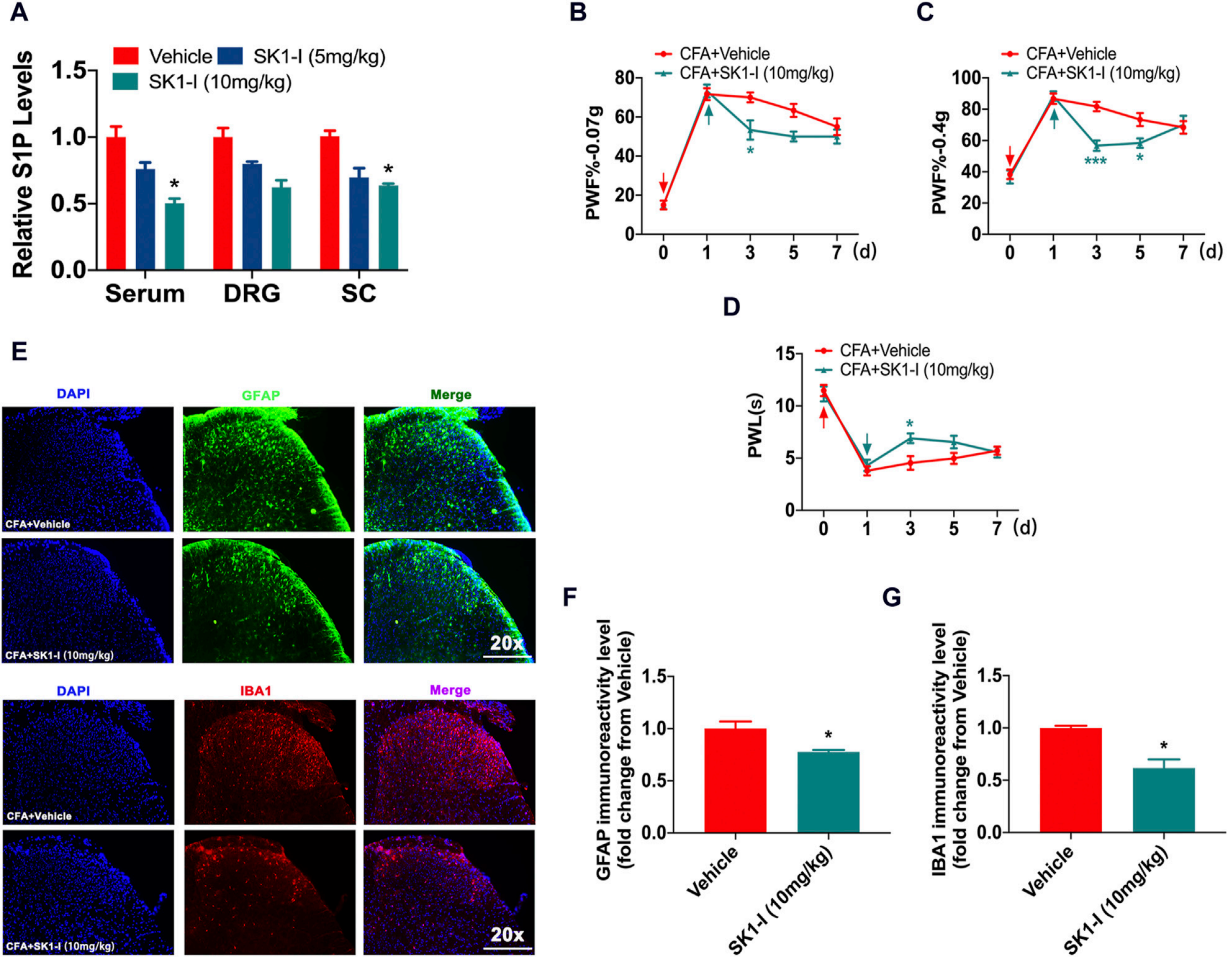
SPHK1 inhibitor reduced nociceptive pain. **(A)** The content of S1P in the serum, DRG and spinal cord of CFA mice on day 3 was analyzed by ELISA after administration of SK1-I (5 mg/kg, 10 mg/kg) (n = 3 biological repeats, 3 mice/repeat/group). Data are shown as the mean ± SEM. *p < 0.05 vs. Vehicle. **(B–D)** The PWF and PWL of CFA mice after intraperitoneal administration of the SPHK1 inhibitor SK1-I (n = 6 mice/group). Data are shown as the mean ± SEM. *p < 0.05, ***p < 0.001 vs. CFA + Vehicle. **(E)** The activation of astrocytes and microglial cells in the spinal dorsal horn was detected by immunofluorescence after SK1-I injection (n = 3–4 biological repeats). **(F,G)** The immunoreactivity levels of GFAP and IBA1 in the spinal dorsal horn after SK1-I administration (n = 3–4 biological repeats). Data are shown as the mean ± SEM. *p < 0.1 vs. CFA + Vehicle. The red arrow represents the time of model establishment. The green arrow represents the time of drug administration.

S1P is a signaling lipid synthesized by sphingosine kinases (SPHK1 and SPHK2) (Jozefczuk et al., 2020; Sukocheva et al., 2020). Since S1P is a pain-inducing substance, does the inhibition of S1P synthesis reduce the nociceptive pain caused by CFA? We found that administration of the SPHK1 inhibitor SK1-I (10 mg/kg) reduced the content of S1P in the serum, DRG and spinal cord in the CFA model (Figure 2A). Intraperitoneal administration of SK1-I (10 mg/kg) reversed the nociceptive pain caused by CFA (Figures 2B–D). SK1-I inhibited the activation of microglial cells and astrocytes in the spinal dorsal horn 3 days after CFA model induction (Figures 2E–G).

### 3.3 S1PR1 was involved in nociceptive pain

**FIGURE 3:**
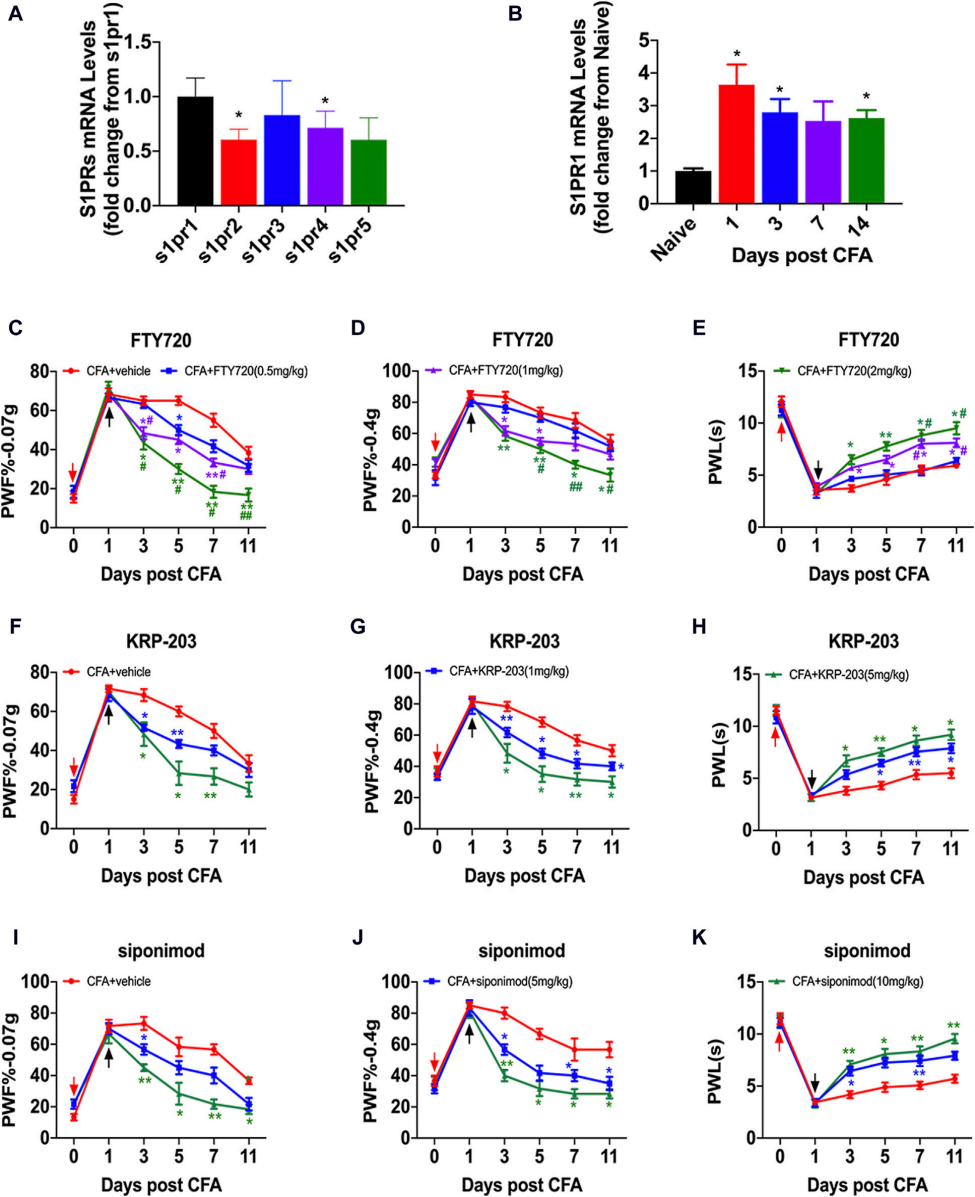
Inhibition of S1PR1 relieved nociceptive pain. **(A)** The content of S1PR mRNA in the spinal cord after CFA was analyzed by qPCR (n = 3 biological repeats, 2–3 mice/repeat/group). Data are shown as the mean ± SEM. *p < 0.05 vs. S1 pr1. **(B)** The level of S1PR mRNA on days 1, 3, 7, and 14 in the spinal cord after CFA model induction (n = 3 biological repeats, 2 mice/repeat/group). Data are shown as the mean ± SEM. *p < 0.05 vs. Naive. **(C–E)** PWF and PWL of mice after i.g. injection of FTY720 (0.5 mg/kg, 1 mg/kg, 2 mg/kg) compared to vehicle on days 0, 1, 3, 5, 7, and 11 after CFA (n = 6 mice/group). **(F–H)** PWF and PWL of mice after i.g. injection of KRP-203 (1 mg/kg, 5 mg/kg) compared to vehicle on days 0, 1, 3, 5, 7, and 11 after CFA (n = 6 mice/group). **(I–K)** PWF and PWL of mice after i.g. injection of siponimod (5 mg/kg, 10 mg/kg) compared to vehicle on days 0, 1, 3, 5, 7, and 11 after CFA (n = 6 mice/group). Data are shown as the mean ± SEM. *p < 0.05, **p < 0.01 vs. vehicle. ^#^ *p* < 0.05, ^##^ *p* < 0.01 vs. FTY720 (0.5 mg/kg). The red arrow represents the time of model establishment. The black arrow represents the time of drug administration.

Previous studies have found that S1P plays a biological role by activating the GPCR signaling pathway through the binding of S1PRs, a homologous receptor on the cell surface. By detecting the mRNA expression of S1PRs in the spinal cord of CFA mice, we found that the mRNA expression level of S1PR1 was significantly increased (Figure 3A). The expression of S1PR1 mRNA was upregulated 1, 3, and 14 days after CFA model establishment (Figure 3B). To further verify the role of S1PR1 in nociceptive pain, we selected several competitive and functional S1PR1 antagonists: FTY720, KRP-203 and siponimod. S1PR1 antagonists were administered daily on day 1 after CFA was established via intragastric (i.g.) administration, and PWF and PWLwere detected on days 0, 1, 3, 5, 7, and 11 of CFA (Figures 3C–K). Irrespective of whether FTY720, KRP-203 or siponimod were provided, PWFs were decreased and PWLs were increased from day 3 after CFA in a dose- and time-dependent manner (Figures 3C–K).

### 3.4 Knockdown of S1PR1 decreased mechanical pain and thermal pain in CFA mice

**FIGURE 4:**
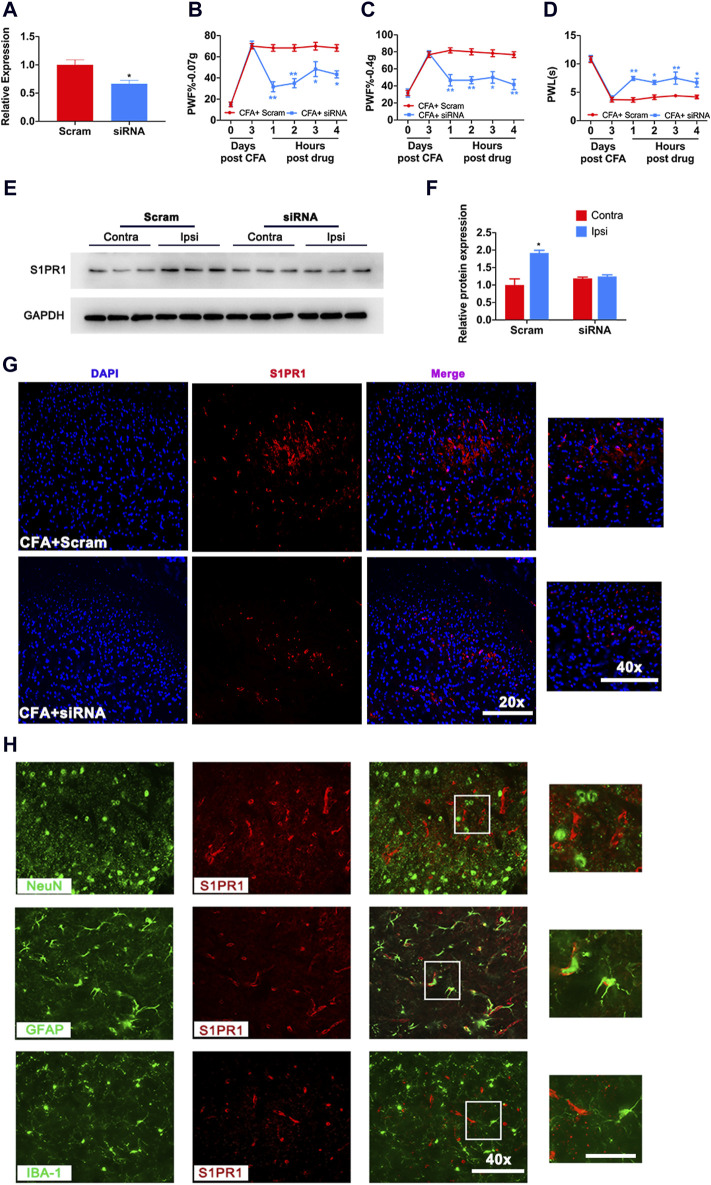
Knockdown of S1PR1 decreased mechanical pain and increased thermal pain in CFA mice. **(A)** The mRNA level of S1PR1 after treatment with S1PR1 siRNA. **(B–D)** PWF and PWL of mice given S1PR1 siRNA compared to Scramble after CFA (n = 3 biological repeats, 2 mice/repeat/group). **(E,F)** The protein expression level after S1PR1 knockdown. **(G)** The expression of S1PR1 in the spinal cord was detected by immunofluorescence after S1PR1 siRNA transfection. **(H)** Localization of S1PR1 with NeuN (the marker of neurons), GFAP and IBA-1 in the spinal cord. Data are shown as the mean ± SEM. *p < 0.05, **p < 0.01 vs. Scramble.

Next, we evaluated mechanical pain and thermal pain in mice after knockdown of S1PR1. The knockdown effect of S1PR1 siRNA was proven by RT‒PCR (Figure 4A). 3 days after CFA, knockdown of S1PR1 via siRNA resulted in markedly decreased PWF and increased PWL, which lasted for 4 h (Figures 4B–D). Western blotting and immunofluorescence also demonstrated that knockdown of S1PR1 reversed the increase in S1PR1 protein levels caused by CFA (Figures 4E–G). Next, we determined the cell location of S1PR1. Immunofluorescence showed that in the CFA model, S1PR1 was specifically expressed in astrocytes, and no obvious colocalization was found in microglia and neurons (Figure 4H).

### 3.5 S1PR1 mediates the activation of STAT3/ERK/p38 MAPK and glial cells after nociceptive pain

**FIGURE 5:**
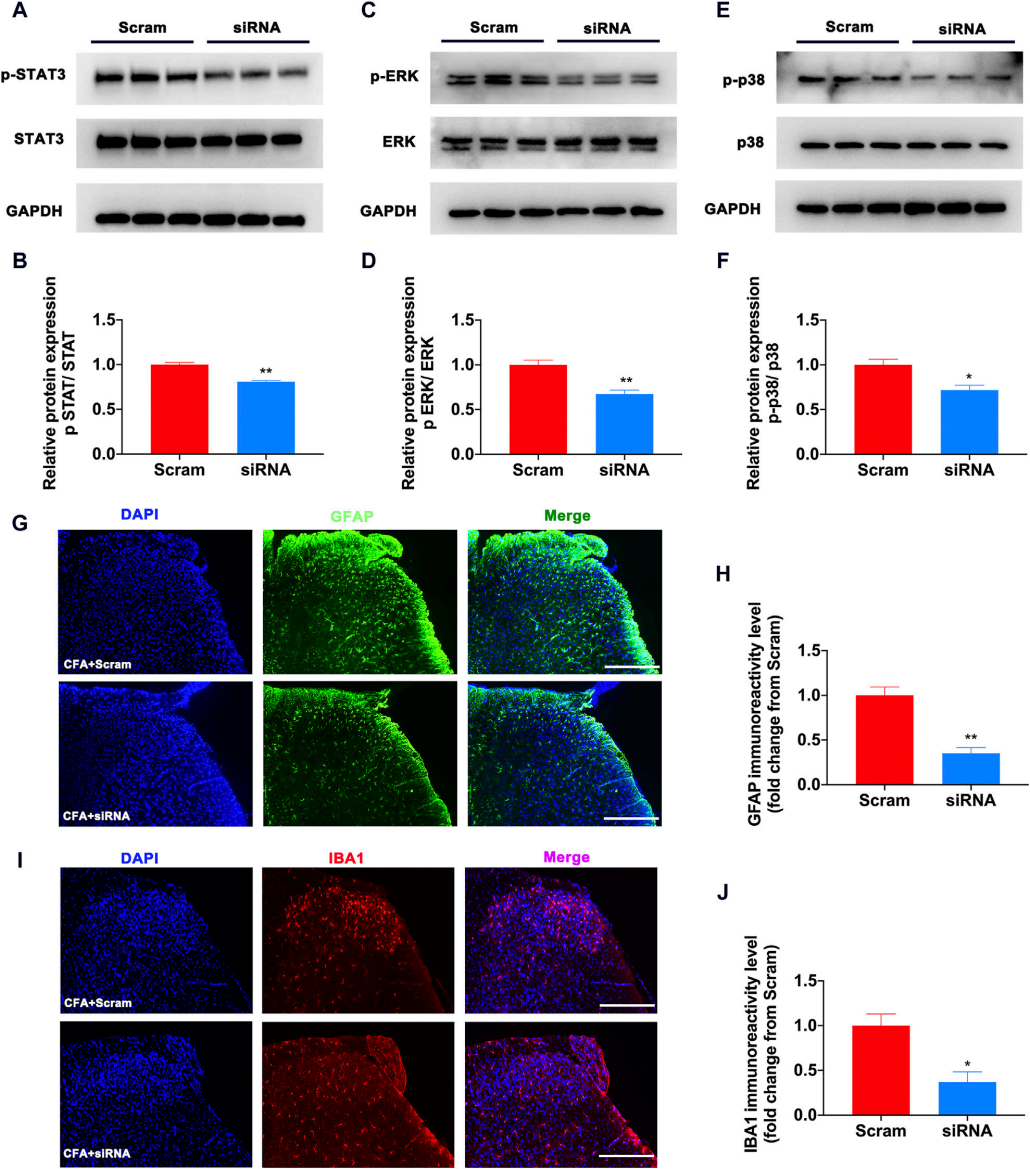
Knockdown of S1PR1 decreased the expression of STAT3/ERK/p38 MAPK and activation of glial cells in CFA mice. **(A,B)** The expression levels of p-STAT3 and STAT3 protein after S1PR1 knockdown (n = 3 biological repeats, 2 mice/repeat/group). **(C,D)** The expression levels of p-ERK and ERK protein after S1PR1 knockdown (n = 3 biological repeats, 2 mice/repeat/group). **(E,F)** The expression levels of p-p38 and p38 MAPK protein after S1PR1 knockdown (n = 3 biological repeats, 2 mice/repeat/group). **(G,H)** The expression of GFAP in the spinal cord was detected by immunofluorescence after S1PR1 siRNA transfection (n = 3–4 biological repeats). **(I,J)** The expression of IBA1 in the spinal cord was detected by immunofluorescence after S1PR1 siRNA transfection (n = 3–4 biological repeats). Data are shown as the mean ± SEM. *p < 0.05, **p < 0.01 vs. Scramble.

To determine why S1PR1 was involved in the development of pain, we combined bioinformatics analysis to identify the proteins that interact with it. The protein‒protein interaction network was created and visualized using Cytoscape. STAT3, ERK, and p38 MAPK have been reported to be important pain-causing molecules (Koichi and Noguchi, 2004; Wan et al., 2017), which suggests that the activation of S1PR1 may activate the downstream pain signaling pathway. Previous studies have found that the activation of STAT3, ERK, and p38 MAPK in glial cells promotes the release of inflammatory factors. Studies on the STAT3 and MAPK families in pain indicated that phosphorylation of these proteins was involved in pain sensitivity caused by inflammation and neuropathic pain (Zhao et al., 2021a; Zhao et al., 2021b). Therefore, we speculate that the activation of S1PR1 leads to the upregulation of the STAT3, ERK, and p38MAPK signaling pathways and participates in the development of pain. On this basis, we verified by Western blotting that the phosphorylation of STAT3, ERK, and p38MAPK decreased significantly after S1PR1 was knocked down (Figures 5A–F). Previous studies have found that activation of glial cell S1PR1 contributes to the development of neuropathic pain, specifically knocking out the S1PR1 allele in GFAP-positive astrocytes, and no SNL-induced neuropathic pain occurs (Chen et al., 2019; Doyle et al., 2020a). Increasing evidence has shown that microglia and astrocytes play an important role in the development of inflammatory pain caused by CFA (Ji et al., 2016). In our study, after using siRNA to inhibit S1PR1, the activation of microglia and astrocytes was significantly inhibited (Figures 5G–J). It is speculated that the activation of S1PR1 on glia is involved in the occurrence of pain.

### 3.6 S1PR1 mediates the activation of inflammatory factors after nociceptive pain

**FIGURE 6:**
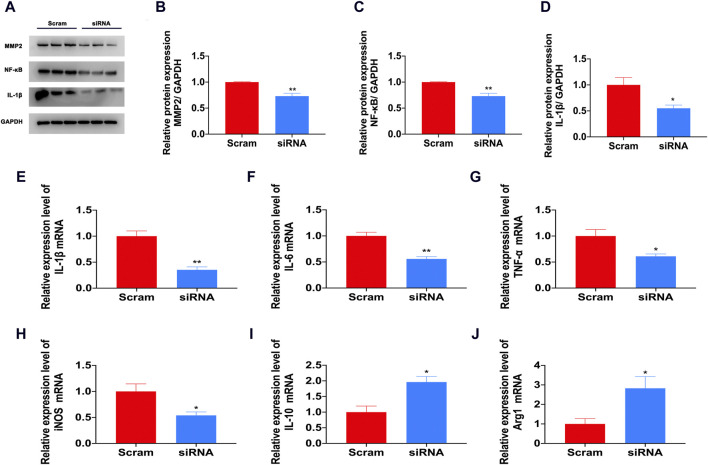
S1PR1 mediated the activation of inflammatory factors in the CFA model. **(A–D)** The protein expression levels of MMP2, NF-κB and IL-1β after S1PR1 knockdown (n = 3 biological repeats, 2 mice/repeat/group). **(E–J)** The mRNA levels of IL-1β **(E)**, IL-6 **(F)**, TNF-a **(G)**, iNOS **(H)**, IL-10 **(I)** and Arg-1 **(J)** in the spinal cord after S1PR1 knockdown (n = 3 biological repeats, 2 mice/repeat/group). Data are shown as the mean ± SEM. *p < 0.05, **p < 0.01 vs. Scramble.

Our previous research found that the expression levels of proinflammatory factors such as IL-1β and TNF-a increased, and the expression level of the anti-inflammatory factor IL-10 decreased. IL-1β and TNF-a are currently widely recognized pain and inflammatory factors that play an important role in the occurrence of neuroinflammation (Pinho-Ribeiro et al., 2017). In the CFA pain model, there was a significant increase in IL-1β mRNA and protein levels (Figures 6A, D, E). Newly generated IL-1β precursors must be hydrolyzed into mature forms by proteases to have pain-inducing effects (Takenouchi et al., 2011; Arman et al., 2020). Recent research shows that during the occurrence and development of pain, the cleavage of IL-1β mainly depends on MMP9 and MMP2 matrix metalloproteinases, and MMP9 cleaves IL-1β to control the earliest stage of pain (<1d). MMP2 cleaves IL-1β, which controls the late stage of development and maintenance of pain (<3d) (Ji et al., 2009). In our study, after inhibiting S1PR1 with siRNA, the expression of MMP2 was inhibited (Figures 6A, B). S1P/S1PR1 induced phosphorylation of NF-κB p65 and activation of NF-κB nuclear translocation (Zheng et al., 2019). We also proved that knockdown of S1PR1 reversed the activation of NF-κB induced by CFA (Figures 6A, C). In addition, the knockdown of S1PR1 inhibited the activation of the inflammatory factors IL-1β, IL-6, TNF-a and iNOS and increased the mRNA levels of the anti-inflammatory factors IL-10 and Arg-1 (Figures 6E–J).

## 4 Discussion

**FIGURE 7:**
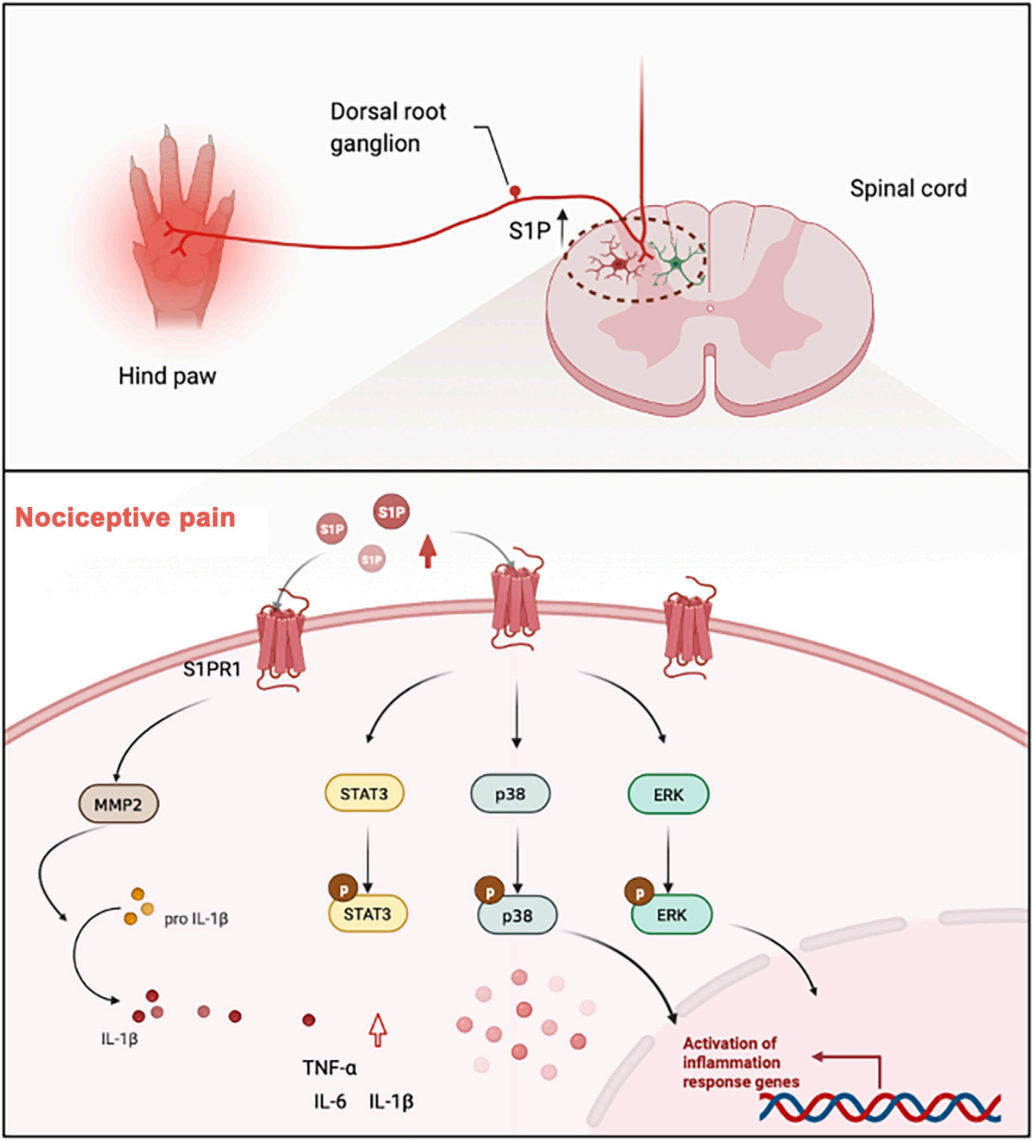
In the model of chronic nociceptive pain caused by CFA, the expression levels of S1P and S1PR1 were significantly increased, which led to the activation of spinal cord glial cells, and S1PR1 activated MMP2 to promote the cleavage of mature IL-1β. The activation of S1PR1 upregulated the phosphorylation of STAT3, ERK and p38 MAPK in glial cells, thus significantly affecting the activation of downstream signaling pathways and ultimately causing inflammatory pain.

The major goal of the current work was to determine whether S1P-S1PR signaling is involved in nociceptive pain, such as inflammatory pain. Our major finding is a marked upregulation in the expression of S1P and S1PR1 in the spinal cord of CFA model mice. We also demonstrated that the S1P-S1PR1 signaling pathway regulated the activation of glial cells in the spinal dorsal horn and the expression of pain-related genes, such as STAT3, ERK, p38MAPK and the inflammatory factors IL-1β, TNF-α, and IL-6. S1PR1 inhibitors or knockdown of S1PR1 could significantly relieve nociceptive pain caused by CFA. This has proven that S1P/S1PR1 may be a new target for pain relief (as is shown in Figure 7).

Sphingomyelin, a natural ingredient in food, has been shown to have strong biological functions in reducing blood lipids, improving skin barrier function, and promoting the development of infant neurons. S1P is one of the metabolites of sphingomyelin and a highly bioactive lipid mediator. S1P has important pathophysiological functions, mainly in inflammation-related diseases and inflammatory responses (Hannun and Obeid, 2008). S1P, a metabolite of sphingomyelin, is a biologically active lipid with important physiological functions that is widely expressed in blood, red blood cells and the central nervous system (Spiegel and Milstien, 2003). Ceramide is converted into S1P by sphingosine kinase SPHK1 and/or SPHK2, and S1P sends signals through the G protein-coupled S1P receptor as an active metabolite (Nielsen et al., 2017; Cartier and Hla, 2019; Langeslag and Kress, 2020). We demonstrate that nociceptive pain elicited alterations in spinal cord sphingolipid metabolism. These changes may represent a novel central inflammatory characteristic. When compared to control animals, CFA mice displayed consistent increases in pro-nociceptive S1P in the ipsilateral spinal cord. S1P contributed to the activation of microglia and astrocytes and the development of hyperalgesia. Pain responses are also triggered by the sphingosine kinase SPHK1 (Langeslag and Kress, 2020).

Previous studies have shown that S1P regulates cell status and neuroinflammation via S1PR1 (Choi et al., 2011). A growing body of evidence suggests that activation of S1PR1 in the spinal cord contributes to the development of mechano-allodynia in a variety of neuropathic pain models, including paclitaxel-induced neuropathic pain and opioid-induced hyperalgesia (Stockstill et al., 2018). Our current study found that S1PR1 was also involved in the occurrence and development of inflammatory pain. The S1PR1 mRNA level was significantly increased in the spinal cord of CFA model mice. In addition, the functional relevance of increases in spinal cord levels of S1PR1 with chronic pain was validated using the S1PR1 functional antagonists FTY720, siponimod or KRP-203. FTY720 is an orally bioavailable highly CNS-permeant (Bolli et al., 2010; Brinkmann et al., 2010) drug that is currently approved for the treatment of relapsing-remitting multiple sclerosis (Harrison, 2014). Knockdown of S1PR1 alleviated mechanical pain and heat pain caused by CFA using siRNA.

Previous studies have demonstrated that S1PR1 is expressed in microglia and astrocytes *in vitro* (Karunakaran et al., 2019; Doyle et al., 2020b). Some studies have shown that blocking S1PR1 signaling promotes the neuroprotective effect of microglia in a variety of central nervous system diseases (Guo et al., 2020). Our study proved that S1PR1 was located in astrocytes in the spinal cord of CFA model mice, which was consistent with a study by Chen et al. (2019). Inhibition of S1PR1 signaling could block the activation of microglia and astrocytes and subsequent release of inflammatory mediators (Pinho-Ribeiro et al., 2017; Karunakaran et al., 2019).

Previous reports demonstrated that S1P induced MMP-9 expression in breast cancer cells and MMP-2 expression in endothelial cells (Kim et al., 2011). Moreover, S1PR1 signaling modulates cell proliferation via MMP-2 (Sassoli et al., 2018). MMP-2 plays important roles in inflammation as well as in pain processes (Kawasaki et al., 2008; Escolano-Lozano et al., 2021). MMP-2 has been proven to contribute to the cleavage of IL-1β and induction of nociceptive pain. In our study, we demonstrated that the CFA model induced upregulation of MMP-2 and IL-1β expression. S1PR1 inhibitors and S1PR1 siRNA inhibited the increase in MMP-2 and IL-1β.

Binding of S1P with S1PR1 can lead to the formation of complexes composed of G-protein or β-arrestin, which are involved in the modulation of the ERK1/2 and p38 MAPK signaling pathways and migration (Zhao et al., 2008; Al-Jarallah et al., 2014; Rostami et al., 2019). We found that inhibition of S1PR1 did indeed reduce the phosphorylation levels of ERK1/2 and p38 in the spinal cord of CFA model mice. S1PR1 is a kind of G-protein-coupled receptor that can induce the production of STAT3 by activating tyrosine kinases and serine/threonine kinases (Pyne and Pyne, 2017).

In conclusion, we have shown the function of the S1P/S1PR1 pathway in CFA model mice. Although the exact role of the S1P pathway in regulating nociceptive pain remains to be fully elucidated, our findings reveal associated molecular pathways that may account for the nociception effect of the S1P/S1PR1 pathway in the CFA model. The results obtained support S1P and S1PR1 as attractive targets for therapeutic nociceptive pain.

## Data Availability

The raw data supporting the conclusions of this article will be made available by the authors, without undue reservation.

## Abbreviations

Arg1, Arginase 1; CFA, Complete Freund’s adjuvant; DAPI, 4′,6-diamidino-2-phenylindole; DRG, dorsal root ganglion; GPCR, G protein coupled receptor; IL, interleukin; iNOS, inducible Nitric Oxide Synthase; PWF, paw withdrawal response frequency; PWL, paw withdraw latency; PBS, phosphate-buffered saline; RT-PCR, Real-time polymerase chain reaction; SM, sphingomyelin; S1P, sphingosine 1-phosphate; S1PR1, sphingosine 1-phosphate receptor 1; SPHK, sphingosine kinases; TNFα, tumor necrosis factor.
